# The longitudinal expression of P. aeruginosa reference genes in infection-mimicking media

**DOI:** 10.1099/mic.0.001627

**Published:** 2026-01-13

**Authors:** Tegan M. Hibbert, Hollie J. Leighton, Sian Pottenger, Daniel R. Neill, Joanne L. Fothergill

**Affiliations:** 1 Department of Clinical Infection, Microbiology and Immunology, University of Liverpool, Liverpool, UK; 2 Division of Molecular Microbiology, School of Life Sciences, University of Dundee, Dundee, UK

**Keywords:** *in vitro *infection models, *Pseudomonas aeruginosa*, quantitative reverse transcription PCR (RT-qPCR), reference genes

## Abstract

Quantitative reverse transcription PCR (RT-qPCR) is a popular and reliable tool for monitoring fluctuations in functional bacterial gene expression. A necessary step of the qRT-qPCR process is the use of a reference gene, which acts to distinguish between technical bias and true biological variation. Many reference genes have been defined for bacterial species; however, few studies have validated their stability across strain types and environmental test conditions. In this study of *Pseudomonas aeruginosa*, the expression consistency of seven commonly used reference genes (*rpoD*, *proC*, *rpoS*, *16S*, *algD*, *gyrA* and *ampC*) was assessed in *P. aeruginosa* laboratory (PAO1) and clinical (LESB65) isolates grown in Lysogeny broth, synthetic cystic fibrosis (CF) media 2 (SCFM2) and CF lung media (CFLM) at various growth time points (2, 6, 24 and 72 h). The stability of the reference genes was then ranked using the RefFinder programme, and three differentially ranked (*rpoS*, *16S* and *ampC*) were used to interpret the expression of a *Pseudomonas* virulence-related gene (*exoS*). The results showed that *16S* was the only reference gene that was quantifiably expressed by both *P. aeruginosa* strains grown in all media types at all growth times. Furthermore, analysing the expression of *exoS* with different reference genes significantly influenced the calculated expression of *exoS* in SCFM2 and CFLM. This study has identified a suitable reference gene for RT-qPCR with *P. aeruginosa* grown in complex respiratory-mimicking media. The results presented here also highlight the importance of validating reference gene expression under the chosen experimental conditions and increase our understanding of how pathogen biology can fluctuate across diverse conditions. Such knowledge is paramount for the development of novel therapeutics, including antimicrobials and anti-virulence agents.

## Introduction

Gene expression analysis is extensively used for an array of purposes, including investigating the transcriptional behaviour of biological systems and classifying cell states in disease [1,2]. Of the techniques available for monitoring fluctuations in functional gene expression, quantitative reverse transcription PCR (RT-qPCR) is amongst the most popular, as it is robust, sensitive and highly reproducible [1,3].

RT-qPCR is a reliable tool for the quantification of bacterial gene expression and can be utilized when sample material is sparse [1]. Furthermore, it is more time-efficient and cost-effective than next-generation sequencing tools when focusing on a small number of target genes [3]. Despite its prominent use, RT-qPCR sample preparation involves several steps that may introduce bias. For example, differences in bacterial RNA extraction efficiencies, the introduction of contaminants during the extraction or cDNA conversion steps or primer mismatching during the RT-qPCR annealing phase [4,5]. To minimize the occurrence of technical bias, an appropriate normalization method is employed to distinguish between true biological variation and that introduced during sample processing [1]. Normalization is typically performed through the employment of reference genes, which are universal internal control genes required for the maintenance of basic cellular function [1,6, 7]. Due to their necessity for cellular homeostasis, bacterial reference genes are assumed to be constitutively expressed across sample types and test environments and conserved across bacterial strains. Although many reference genes have been defined for bacterial species, there is a lack of consensus as to which might be most reliable as stably expressed internal controls. Few studies validate consistency of reference gene expression across test environments or strains used. This is the case even for well-studied bacterial species, such as *Pseudomonas aeruginosa* [8,9]. *P. aeruginosa* is a common laboratory model to study processes such as biofilm formation and quorum sensing. It is also well studied due to its roles in accelerating the decline of pulmonary function in cystic fibrosis (CF) lung infections [10]. A few studies have indicated that the expression of common *P. aeruginosa* reference genes can fluctuate under certain experimental conditions [1,11]. These are important observations, as if similar fluctuations occur across respiratory infection sites or across an infection time course, there are implications for analysis and interpretation of RT-qPCR gene expression data collected under conditions designed to mimic infection environments.

In this study, SYBR Green RT-qPCR was utilized to quantify longitudinal changes in the expression of seven commonly used *P. aeruginosa* reference genes during bacterial growth in complex environments. For this, we used a common laboratory medium and two sputum mimics designed to capture the chemical conditions of the CF airways: synthetic CF media 2 (SCFM2) and CF lung media (CFLM) [12,13]. The selected reference genes were *rpoD* [14], *proC* [14], *rpoS* [14], *16S* [15], *algD* [16], *gyrA* [16] and *ampC* [14]. These encode a range of genes associated with sigma factors, protein synthesis and DNA replication. The bacterial growth time points assessed were 2, 6, 24 and 72 h, and no template controls were included in the RT-qPCR reactions.

The stability of these seven classic *P. aeruginosa* reference genes across bacterial growth phases and between strains was investigated. The aim was to identify a suitable reference gene with strain, growth phase and environment-independent expression. Such reference genes could therefore be employed in assessing the longitudinal expression of *P. aeruginosa* genes across a variety of environmental niches.

## Methods

### Selection of reference genes and design of primer pairs

Seven genes (*rpoD*, *proC*, *rpoS*, *16S*, *algD*, *gyrA* and *ampC*) were selected for use in this study. These genes have been used as * P. aeruginosa* reference genes in several studies [14,16]. The *proC* and *rpoD* primer pairs were identified from a study by Fothergill *et al.* [17]. With reference to the whole-genome sequence of *P. aeruginosa* PAO1 (accession: NC 002516), the *rpoS*, *16S*, *algD*, *gyrA* and *ampC* primers were designed and manufactured using National Center for Biotechnology Information (NCBI)-primer-blast [18]. During manufacture, primers were purified using the High Purify Salt Free method. The list of primers used in this study, along with the gene functions, can be found in Table S1, available in the online Supplementary Material. Primer selection, RNA extraction, RT-qPCR assays and subsequent data analysis were conducted in accordance with the MIQE guidelines to ensure methodological rigour and data reliability.

### Bacterial growth conditions

*P. aeruginosa* PAO1 and LESB65 (accession: CP006983) strains were grown overnight as three biological replicates in Lysogeny broth (LB) at 37 °C with shaking at 180 r.p.m. [19,20]. OD_600_ readings were taken and adjusted to 0.07–0.1. Next, 1 ml of overnight culture was centrifuged for 5 min at 14,800 r.p.m., and the supernatant was removed. The bacterial pellet was resuspended in 20 ml test media (LB, SCFM2, CFLM) and incubated for 2, 6, 24 and 72 h at 37 °C with shaking at 180 r.p.m. SCFM2 was prepared as described by Turner *et al.* [13]. CFLM was prepared as described by Ruhluel *et al.* [12]. Importantly, 10 µl of sterile test media was taken from the original stock, and Miles and Misra plates were performed to ensure sterility of the media prior to the addition of the pellet. No growth was observed from any of the test media. Bacterial concentrations of PAO1 and LESB65 at each time point and in each media type are presented in Fig. S3.

### RNA extraction and cDNA synthesis

RNA was extracted immediately after the required incubation using the Direct-zol RNA Miniprep (Zymo Research), with no fixing required. The manufacturer’s protocol was optimized to increase RNA yield: multiple bacterial pellets were taken from the inoculated test media after incubation by centrifuging 1 ml culture for 5 min at 1,480 r.p.m. and removing the supernatant (for 2 and 6 h, 12 pellets per biological replicate were used; for 24 and 72 h, 8 pellets per biological replicate were used). During RNA purification step 1: pellets in SCFM2 and CFLM were manually disrupted with a 0.2–0.6 mm needle 5–10 times or until the pellet was completely disrupted after the addition of TRI reagent. A DNase treatment was performed according to the manufacturer’s guidelines. The RNA was eluted in 50 µl ribonuclease-free water, and the concentration was quantified using the Qubit 4 Fluorometer and the Qubit Broad Range assay kit (ThermoFisher Scientific). RNA purity was quantified using the NanoDrop spectrophotometer (NanoDrop Technologies Inc.). The A_260/280_ and A_260/230_ wavelength results are reported in Table S4. The NanoDrop spectrophotometer was also used to assess for RNA purity (A260/A280). After the completion of RT-qPCR experimentation, RNA was stored at −80 °C.

cDNA was synthesized using the iScript cDNA Synthesis Kit (Bio-Rad). Per reaction, 4 µl 5× iScript Reaction Mix, 1 µl iScript Reverse Transcriptase, 14 µl ribonuclease-free water and 1 µl RNA were used. The cDNA synthesis conditions were as follows: 5 min at 25 °C for 5, 30 min at 42 °C and 5 min at 85 °C. Wherever possible, 50 ng µl^−1^ of RNA was used for cDNA synthesis. cDNA was stored at −20 °C. The concentrations of RNA used for cDNA synthesis for each media type and time point can be found in Table S4.

### RT-qPCR assay

SYBR Green RT-qPCR was manually performed using GoTaq qPCR Master Mix (Promega) and conducted on the Rotor-Gene Q (Qiagen) using 0.1 ml 4-Strip Rotor-Gene Style Tubes and Caps (StarLab). The reaction mix included 2 µl cDNA, 10 µl GoTaq qPCR Master Mix (Promega), 1 µl forward primer and 1 µl reverse primer (diluted to give a final concentration of 4 µM) and 6 µl ribonuclease-free water, to give a final volume of 20 µl. The RT-qPCR conditions were as follows: 2 min at 95 °C (initial denaturation), 40 cycles of 15 s at 95 °C (denaturation) and 1 min at 60 °C (annealing/fluorescent acquisition). The melting temperature-determining dissociation step was performed to assess for the presence of RNA and DNA contamination and the secondary structures, and conditions were as follows: 95 °C for 15 s and 60 °C for 1 min and 95 °C for 15 s at the end of the amplification. An example melt curve is shown in Fig. S1. Cycle threshold (*Ct*) values were determined by the Rotor-Gene ScreenClust HRM Software – VP6 JS (Qiagen) through the incorporation of two standards of known RNA concentration [PAO1 (LB) 50 and 5 ng µl^−1^]. RT-qPCR reactions were carried out in biological triplicate (excluding the qPCRs performed for the expression analyses of *exoS*, which were performed in biological quadruplicate) for each cDNA sample. No template controls were performed using ribonuclease-free water and also in triplicate. Technical triplicates were also performed for each reaction. Prior to the performance of test RT-qPCRs, reference gene primer standard curves were performed using *P. aeruginosa* PAO1 gDNA extracted using Quick DNA MiniPrep kit (Zymo). *Ct* values, standard curve graphs and primer efficiencies can be found in Tables S5 and S6 and Fig. S2. It should also be noted that all RNA extractions, cDNA synthesis and qPCR reactions were performed in the host laboratory at the University of Liverpool.

### Reference gene expression stability analysis

The expression stability of the seven selected reference genes was analysed using RefFinder, a web-based comprehensive tool developed for evaluating reference genes from a given dataset [21,22]. It utilizes several major computational programmes (geNorm, NormFinder, BestKeeper and the Δ-*Ct* method) to rank reference genes based on their stability across a variety of conditions [23,26]. The mean value of the RT-qPCR reaction triplicates was calculated for each condition and then input into the RefFinder application. BestKeeper provides two indicators for reference gene stability, raw sd from the *Ct* values and the coefficient of correlation (*r*) based on each of the reference genes, as calculated from the geometric mean of the remaining genes [24,27]. Typically, reference genes with an sd >1 are deemed unstable, whereas reference genes with *r* values close to 1 are considered the most stable [24,27]. NormFinder calculates stability through calculating both the intra- and inter-group variation and then combines the values and finds the square root. geNorm calculates all possible pairwise ratios between genes exposed to the same condition to give an expression ratio [26]. The sd of the log-transformed value is then calculated for each ratio to determine variability in paired reference genes across test conditions [26]. A low sd suggests that the two genes in the given ratio maintain a consistent expression ratio. geNorm then determines the stability (*M* value) for each reference gene [26]. The *M* value is the average sd of the expression ratios between a given reference gene and the other reference genes within the dataset. NormFinder and geNorm set the stability cut-off value at 0.15, with geNorm *M* values below 0.15 indicating the inclusion of an additional reference gene is not required [23,26].

RefFinder calculates the geometric mean through collecting the stability scores for each reference gene as calculated by each algorithm, multiplying them and then calculating the *n*th root, where *n* is the number of stability scores [22].

## Results

### Expression of reference genes across different time points and media types

**Fig. 1.**
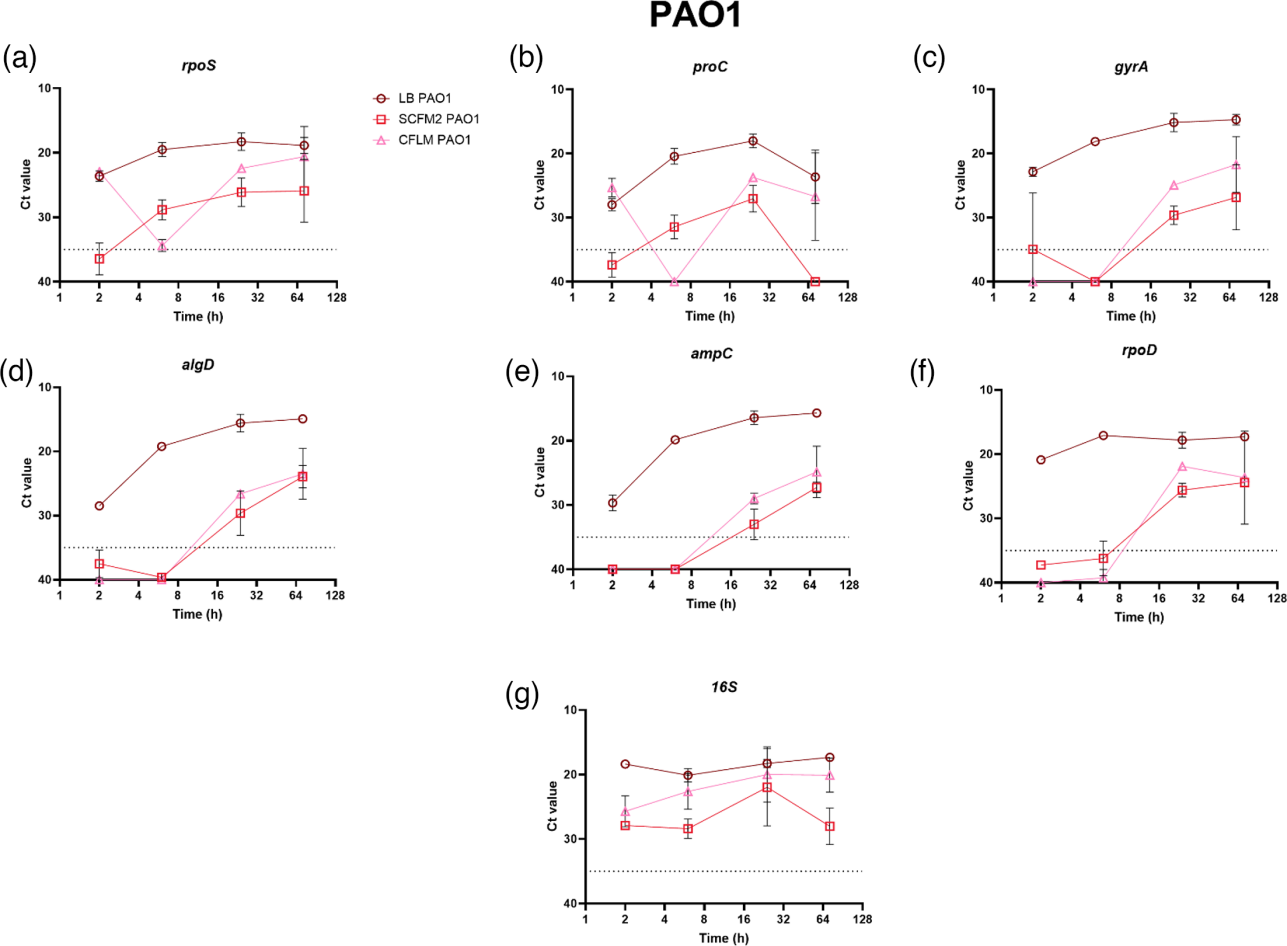
The *Ct* values of seven genes in *P. aeruginosa* PAO1 grown in LB, SCFM2 and CFLM at 2, 6, 24 and 72 h. Three biological replicates and three technical replicates were performed for each condition. SYBR Green qPCR was used to determine *Ct* values. *Ct* values ≥35 or ≤5 were deemed unsatisfactory, as *Ct* values outside of these limits may be the result of non-specific amplification. The 35 *Ct* cut-off value is defined on the graphs as a dashed line. (**a**) *rpos*, (**b**) *proC*, (**c**) *gyrA*, (**d**) *algD*, (**e**) *ampC*, (**f**) *rpoD* and (**g**) *16S*. Using GraphPad Prism 10.0.0 for Windows, GraphPad Software, Boston, Massachusetts, USA, https://www.graphpad.com/.

The longitudinal expression profiles of seven reference genes of PAO1 grown in LB, SCFM2 and CFLM are shown in Fig. 1. When PAO1 was grown in LB, the *Ct* values of all the reference genes across the time points fell within the determined acceptable range (≤35 *Ct*≥5). *Ct* values outside of this range are regarded as unreliable, often due to non-specific amplification, and are generally removed from analysis [28]. The expression of the reference genes was less consistent across the time points when PAO1 was grown in SCFM2 and CFLM, particularly for *rpoS*, *proC*, *gyrA*, *ampC*, *algD* and *rpoD* (Fig. 1a–f), with all demonstrating *Ct* values greater than the 35-cycle threshold cut-off for at least one time point. *16S* was the only reference gene which demonstrated *Ct* values within the determined range across 2, 6, 24 and 72 h for PAO1 grown in all media types (Fig. 1g).

**Fig. 2.**
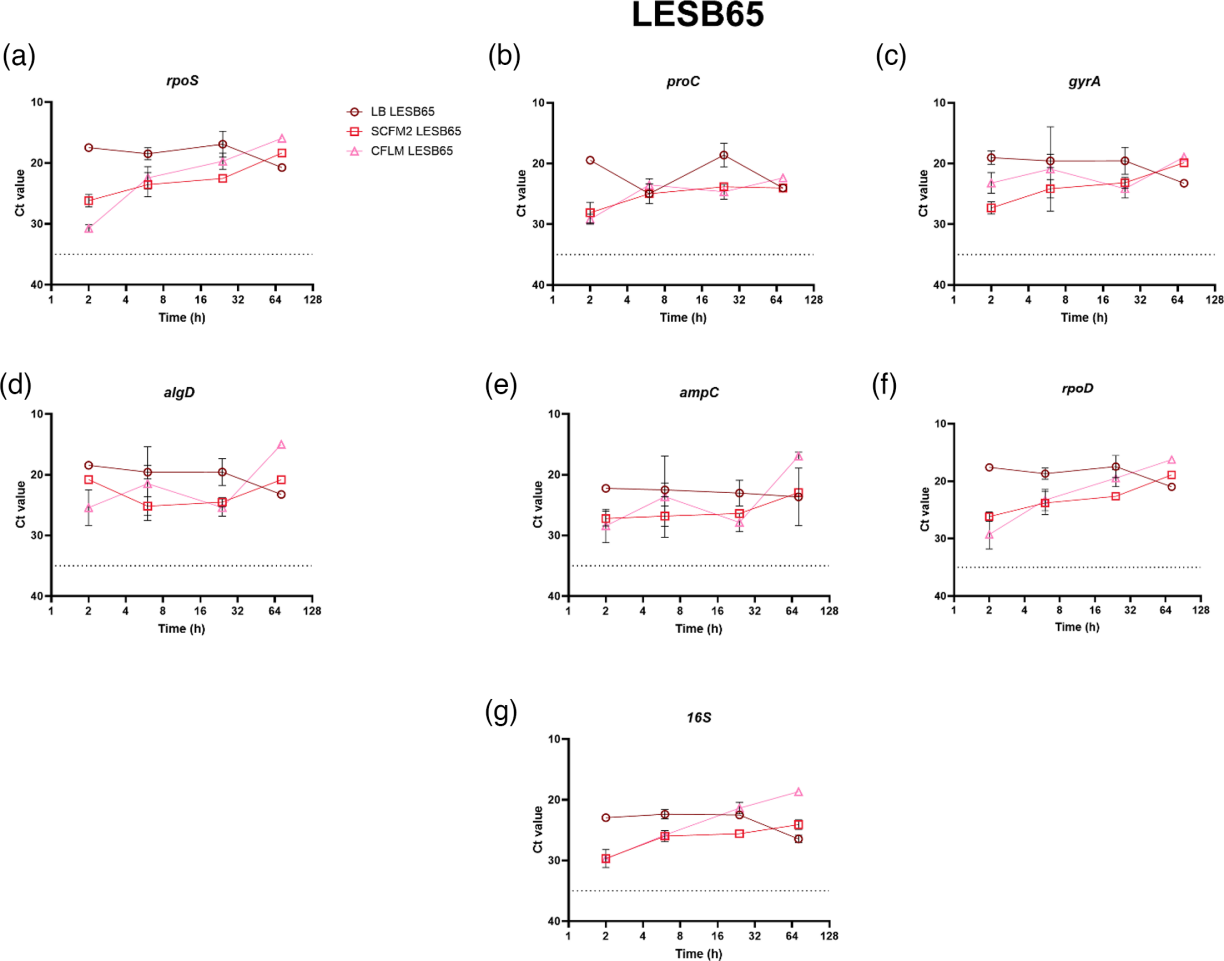
The *Ct* values of seven genes in *P. aeruginosa* LESB65 grown in LB, SCFM2 and CFLM at 2, 6, 24 and 72 h. Three biological replicates and three technical replicates were performed for each condition. SYBR Green qPCR was used to determine *Ct* values. *Ct* values ≥35 or ≤5 were deemed unsatisfactory, as *Ct* values outside of these limits may be the result of non-specific amplification. The 35 *Ct* cut-off value is defined on the graphs as a dashed line. (**a**) *rpos*, (**b**) *proC*, (**c**) *gyrA*, (**d**) *algD*, (**e**) *ampC*, (**f**) *rpoD* and (**g**)*16S*. Using GraphPad Prism 10.0.0 for Windows, GraphPad Software, Boston, Massachusetts, USA, https://www.graphpad.com/.

Conversely, the expression profile of the seven reference genes of LESB65 grown in SCFM2 and CFLM was much more consistent across the time points than seen for PAO1 when grown in the corresponding media (Fig. 2). The *Ct* values of all the reference genes at 2, 6, 24 and 72 h also fell within the acceptable range for LESB65 grown in LB (Fig. 2). The differences in expression profile seen between the two strains could be due to the fact that LESB65 is a clinical isolate that is already adapted to the lung environment. The raw *Ct* values of the reference genes for both PAO1 and LESB65 can be found in Tables S2.1–S2.3. To ensure that the gene expression results observed at 2 h were not influenced by residual effects of overnight growth in LB, RT-qPCRs were also performed to assess *16S* expression in PAO1 and LESB65 following overnight culture in the test media, with subsequent incubation in fresh test media for 2 h. No significant differences in *16S* expression were observed between cultures grown overnight in LB or the test media, as is presented in Tables S7 and S8.

### Expression stability analysis by Delta-*Ct*, BestKeeper, NormFinder and geNorm

To evaluate the stability of the candidate reference genes, the four most popular algorithms for assessing reference gene stability, BestKeeper [24], Delta-*Ct* [25], geNorm [26] and NormFinder [23], were applied, utilizing the web-based tool, RefFinder [21,22]. RefFinder combines each algorithm to calculate a geomean for every candidate reference gene, ranking them accordingly.

**Fig. 3.**
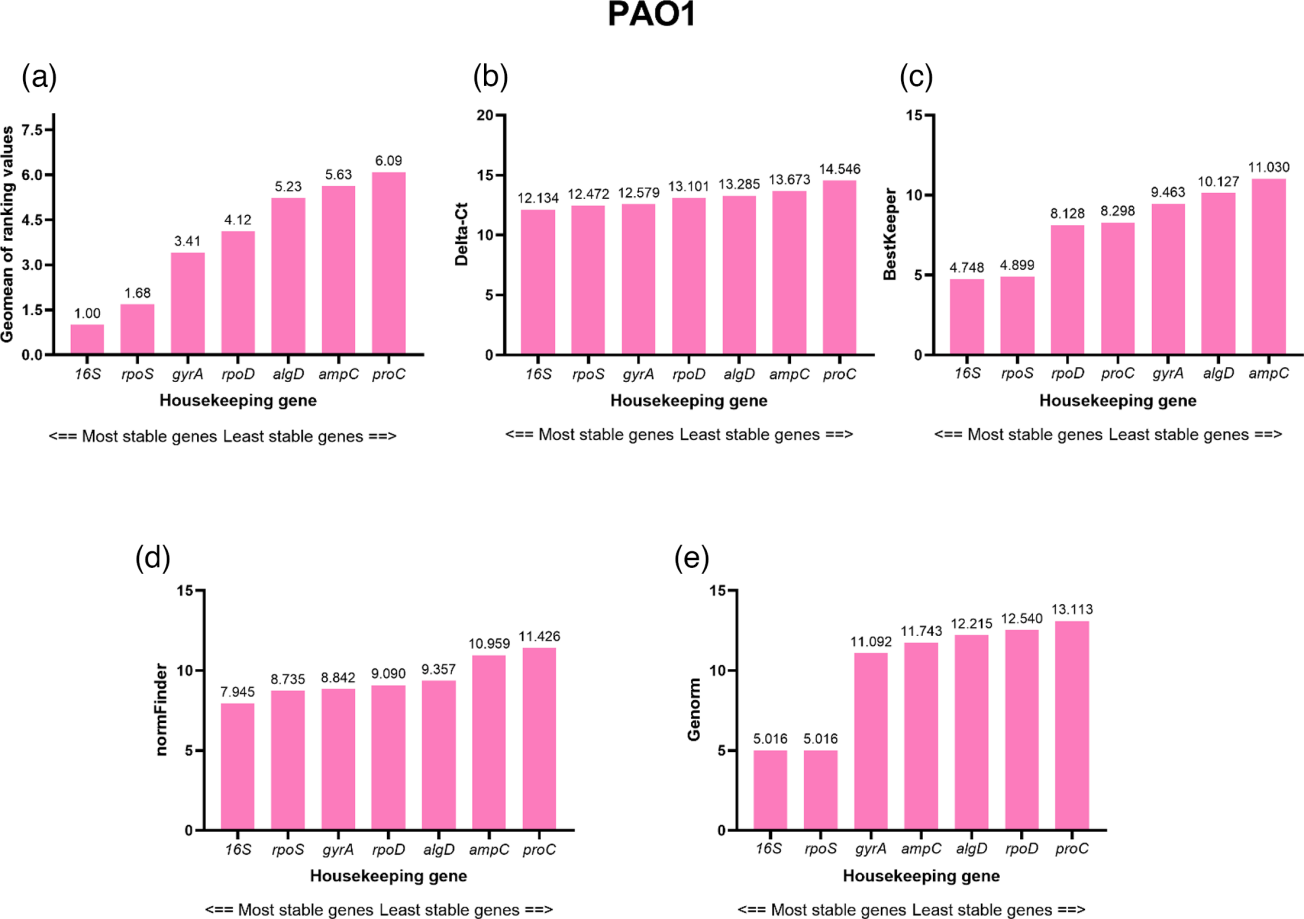
Results of analysing selected housekeeping gene expression in *P. aeruginosa* PAO1 using the RefFinder online tool. (a) RefFinder calculates a comprehensive geomean stability value using stability calculations from (b) Delta-*Ct*, (c) BestKeeper, (d) NormFinder and (e) geNorm. Using GraphPad Prism 10.0.0 for Windows, GraphPad Software, Boston, Massachusetts, USA, https://www.graphpad.com/.

The stability of the reference genes for PAO1, incorporating all media types and time points, is shown in Fig. 3. *16S* was ranked the most stable when evaluated by all four algorithms. However, despite its stability ranking, *16S* exceeded the acceptable parameters set by several of the algorithms (Fig. 3c–e): the stability ranking of *16S* was 4.748 utilizing BestKeeper (Fig. 3c). Despite being ranked most stable by both algorithms, *16S* also exceeded the stability values for the NormFinder (Fig. 3d) and geNorm (Fig. 3e) algorithms, at 7.945 and 5.016, respectively. However, it must be noted that the stability values for the RefFinder algorithms were designed using data from eukaryotic studies; therefore, the applicability of the described thresholds in assessing prokaryotic reference gene stability is unknown.

**Fig. 4.**
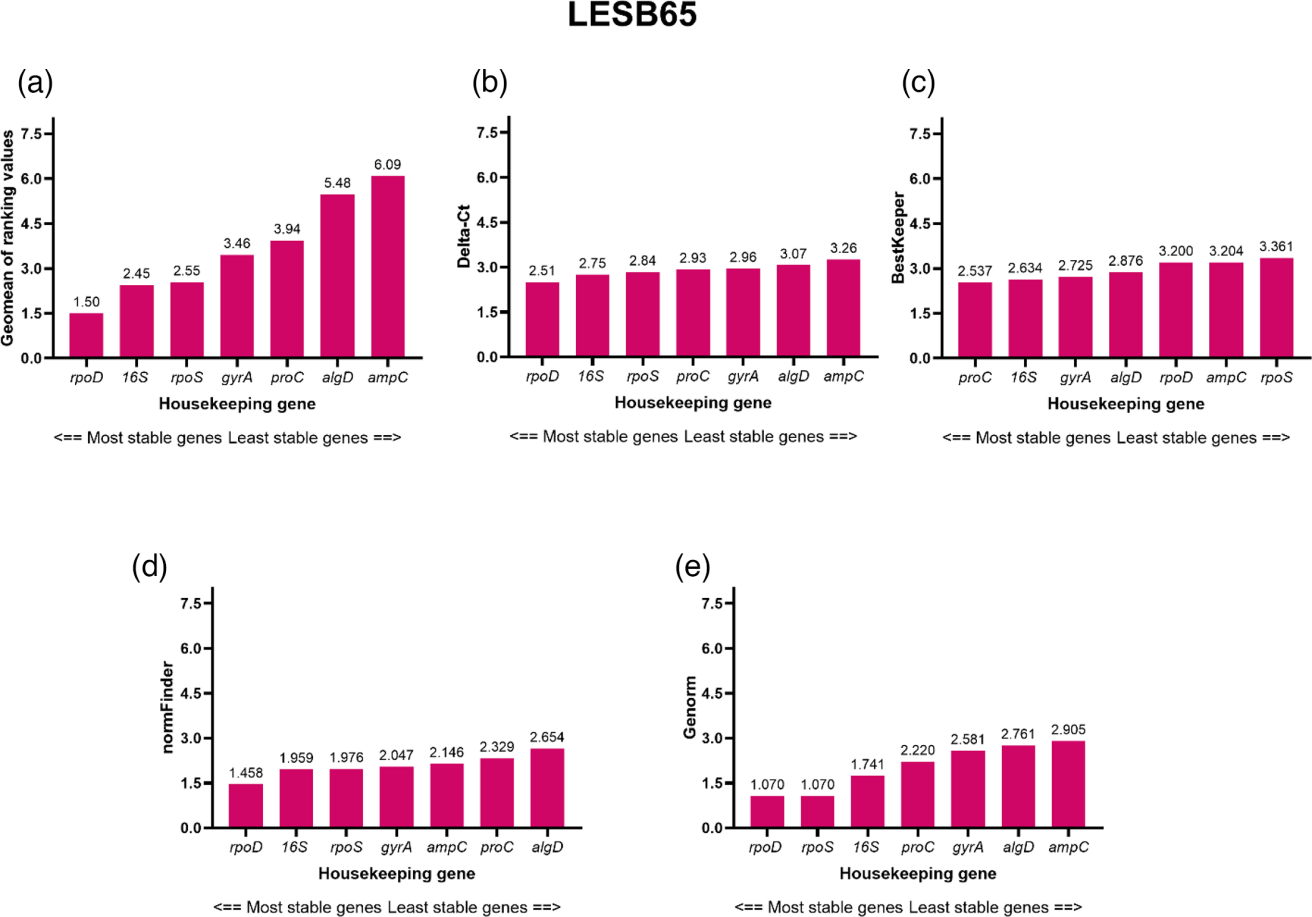
Results of analysing selected housekeeping gene expression in *P. aeruginosa* LESB65 using the RefFinder online tool. (a) RefFinder calculates a comprehensive geomean stability value using stability calculations from (b) Delta-*Ct*, (c) BestKeeper, (d) NormFinder and (e) geNorm. Using GraphPad Prism 10.0.0 for Windows, GraphPad Software, Boston, Massachusetts, USA, https://www.graphpad.com/.

The stability of the reference genes for LESB65 is shown in Fig. 4. Although not ranked as stable as in PAO1, *16S* was ranked in the top three most stable reference genes for all the algorithms and was ranked the second most stable reference gene (behind *rpoD*) in the comprehensive gene stability ranking for LESB65 (Fig. 4). This highlights *16S* as the highest overall ranking reference gene across both genetic backgrounds. However, despite its positioning in the stability analysis rankings, *16S* also exceeded the stability parameters set by several of the algorithms for LESB65 (Fig. 4c–e).

### Expression stability analysis ranked dependent on time point and media type

**Fig. 5.**
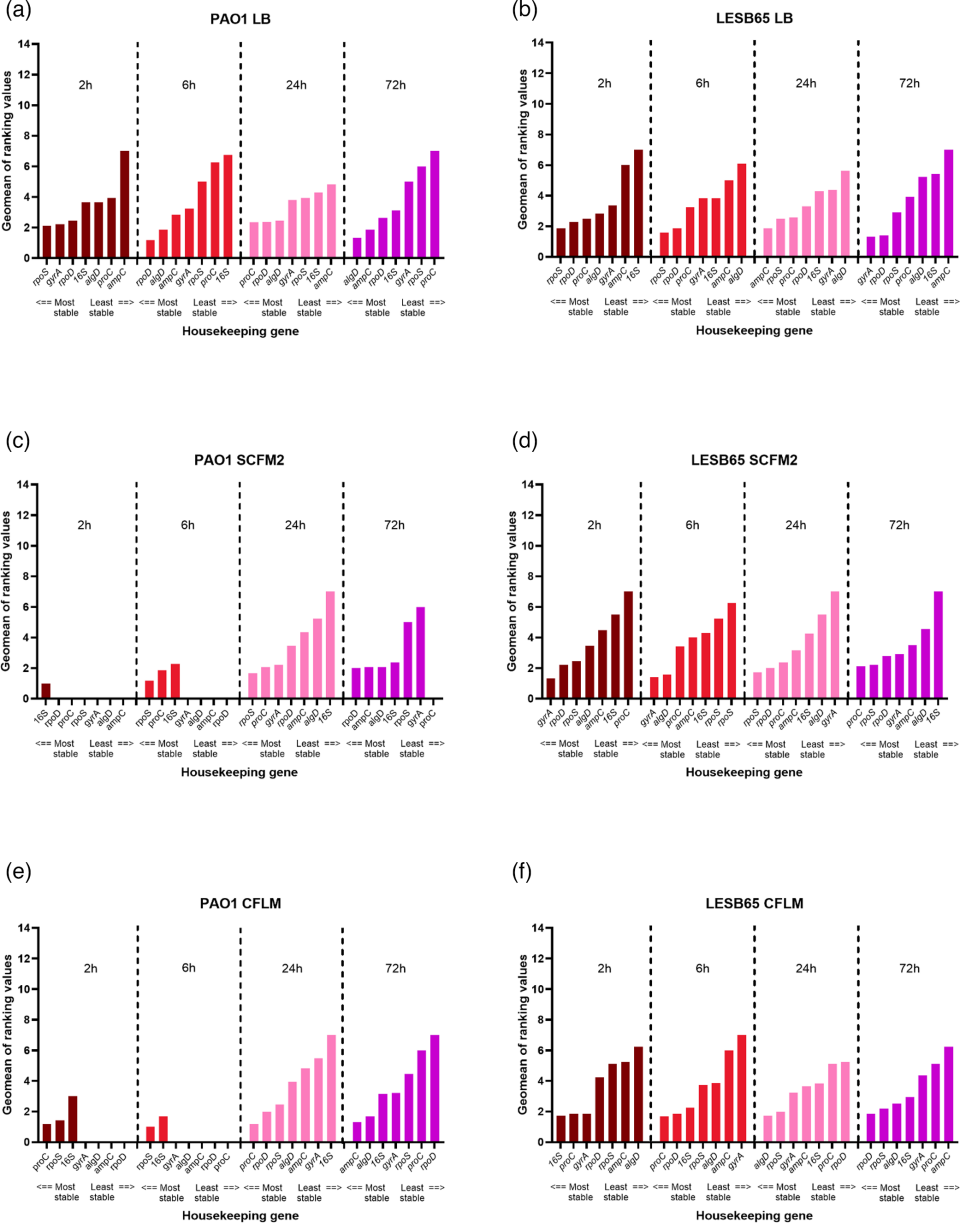
Analysis of reference expression in *P. aeruginosa* PAO1 and LESB65 across media type and time point. The RefFinder online tool was used to rank the reference expression in PAO1 and LESB65 grown in LB, SCFM2 and CFLM at 2, 6, 24 and 72 h following inoculation. Values at 0 indicate no expression of that gene at the given time point. (**a**) PAO1 LB, (**b**) LESB65 LB, (**c**) PAO1 SCFM2, (**d**) LESB65 SCFM2, (**e**) PAO1 CFLM and (**f**) LESB65 CFLM. Graphs produced using GraphPad Prism 10.0.0 for Windows, GraphPad Software, Boston, Massachusetts, USA, https://www.graphpad.com/.

To better evaluate the stability patterns of the reference genes, the ranking analysis was performed again for each strain, this time separated by media type and time point (Fig. 5). The stability profile of the reference genes varied depending on the growth conditions, with differences observed across the media types within the same time point, suggesting that environment influences *P. aeruginosa* reference gene expression. Despite not consistently ranking as most stable across media types and time points for either PAO1 or LESB65, *16S* was the only reference gene which was expressed within the determined range for all the conditions evaluated (Fig. 5).

### Expression analysis of *exoS* using differentially ranked reference genes

**Fig. 6.**
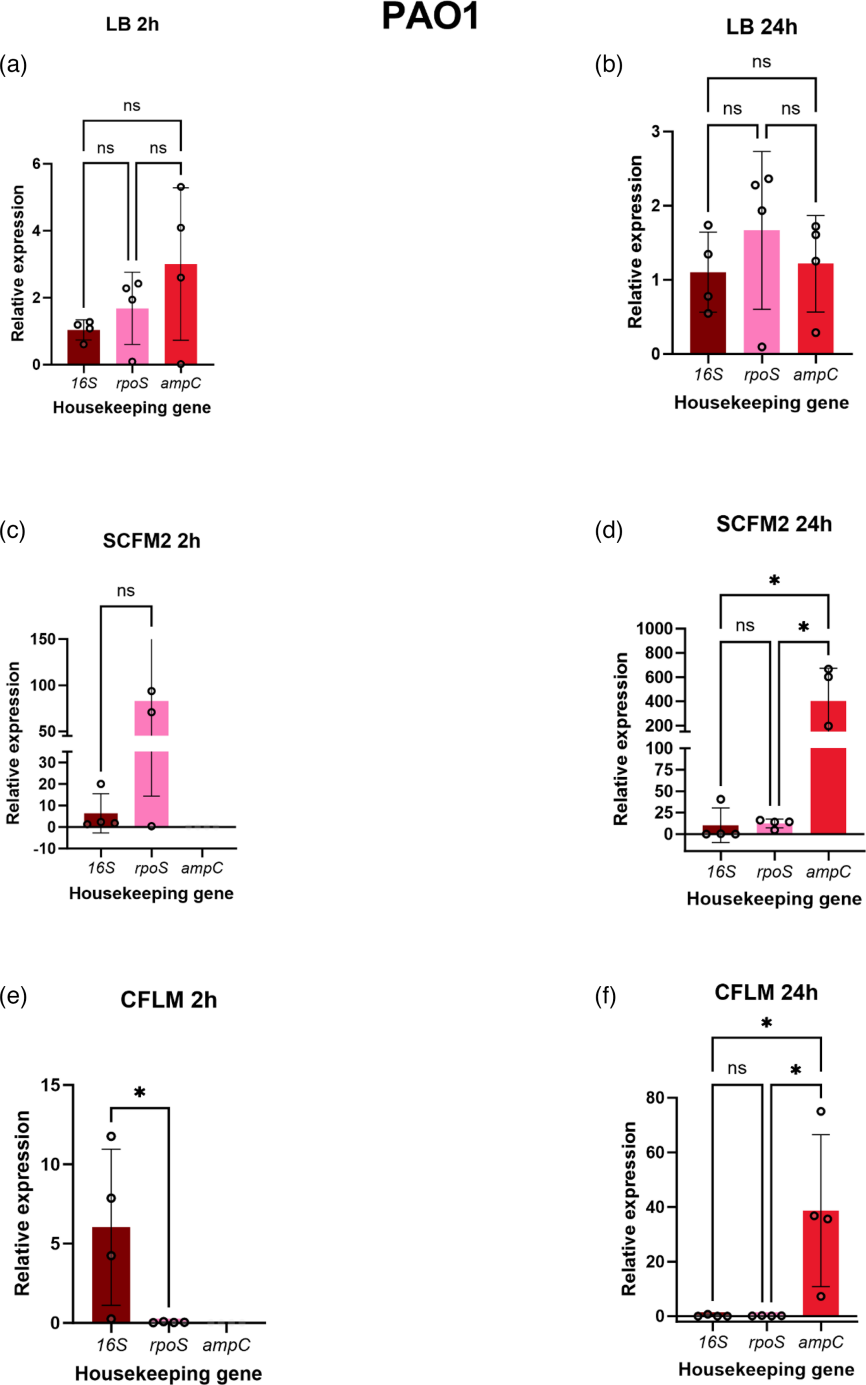
The comparison of analysing *exoS* expression in *P. aeruginosa* PAO1 at 2 and 24 h in LB, SCFM2 and CFLM using *16S*, *rpoS* and *ampC*. Analysed using the 2-∆∆*Ct* method. (**a**) LB 2 h, (**b**) LB 24 h, (**c**) SCFM2 2 h, (**d**) SCFM2 24 h, (**e**) CFLM 2 h and (**f**) CFLM 24 h. One-way ANOVA followed by Tukey’s multiple comparison test was performed using GraphPad Prism 10.0.0 for Windows, GraphPad Software, Boston, Massachusetts, USA, https://www.graphpad.com/. *P≤0.05, **P≤0.01, ns=not significant.

**Fig. 7.**
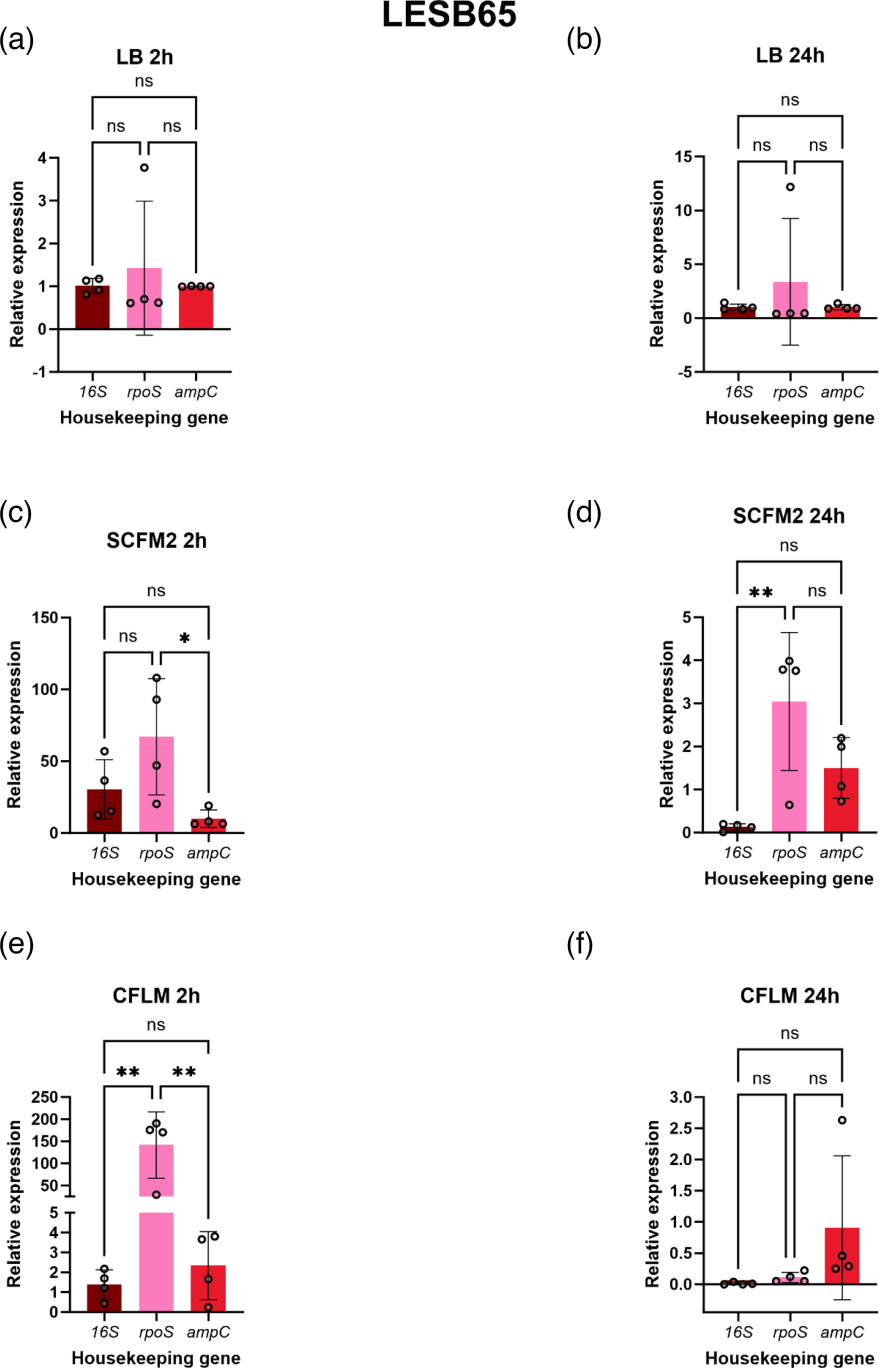
The comparison of analysing *exoS* expression in *P. aeruginosa* LESB58 at 2 and 24 h in LB using *16S*, *rpoS* and *ampC*. Analysed using the 2-∆∆*Ct* method. (**a**) LB 2 h, (**b**) LB 24 h, (**c**) SCFM2 2 h, (**d**) SCFM2 24 h, (**e**) CFLM 2 h and (**f**) CFLM 24 h. One-way ANOVA followed by Tukey’s multiple comparison test was performed using GraphPad Prism 10.0.0 for Windows, GraphPad Software, Boston, Massachusetts, USA, https://www.graphpad.com/. *P≤0.05, **P≤0.01, ns=not significant.

To understand how the stability ranking of the reference genes can affect the analysis of bacterial virulence gene expression, three differentially ranked reference genes (*16S*, *rpoS* and *ampC*) were used to analyse the expression of *exoS* in both strains in LB, SCFM2 and CFLM after 2 and 24 h of growth using the traditional 2-∆∆*Ct* method, with ∆*Ct* of *exoS* in LB used as the control in the calculation (Figs 6 and 7 and Table S3). These reference genes were selected as they were the only genes expressed at 2 and 24 h in all media types. Exoenzyme S (exoS) is one of four effector proteins that are secreted by the *P. aeruginosa* type III secretion system and contributes to pathogenicity through interference with host cell signalling pathways, driving apoptosis and tissue damage [29,30]. When grown in LB, there was no significant difference in *exoS* expression when analysed with normalization against the three reference genes at either time point for both strains (Figs 6 and 7). However, significant differences in *exoS* expression were observed for PAO1 and LESB65 grown in the complex media types when normalized against different reference genes. For PAO1 grown for 24 h in SCFM2 (Fig. 6d), the expression of *exoS* when normalized against *ampC* was significantly higher than when normalized using *16S* or *rpoS*, at a relative expression of 401.6-fold, compared to 10.27 for *16S* and 12.31 for *rpoS* (*16S* vs. *ampC P*=0.0162, *rpoS* vs. *ampC P*=0.0166). A large variation in *Ct* values was also seen for *exoS* expression when normalized against *ampC* in this condition. Conversely, when PAO1 was grown in CFLM for 2 h, no comparisons could be made between *exoS* expression when normalized with *ampC*, due to the lack of *ampC* expression by PAO1 at this time point in CFLM (Fig. 6e). However, *exoS* expression was significantly higher when normalized with *16S* compared to *rpoS* under these conditions (*P*=0.0374)*.* It must be noted that, when PAO1 is grown in CFLM for 2 h, there is a much wider distribution of data amongst the biological repeats in *exoS* analysed with *16S* than seen when the exotoxin gene was analysed with *rpoS*. There were also significant differences in *exoS* expression when normalized with the different reference genes for LESB65 grown in the complex media types (Fig. 7). For LESB65 grown in SCFM2 for 2 h (Fig. 7c), a significant increase in *exoS* expression was observed when normalized with *rpoS*, in comparison with *ampC*. However, the expression of *exoS* when normalized by *rpoS* under these conditions displayed wide variability across biological repeats. The expression of *exoS* was lowest when normalized by *16S* in LESB65 grown in SCFM2 for 24 h and was significantly lower than when *rpoS* was used for normalization under these conditions (*16S* vs. *rpoS P*=0.0071) (Fig. 7d)*.* The expression of *exoS* was also significantly lower when normalized by *16S* compared with *rpoS* in LESB65 grown in CFLM for 2 h (*16S* vs. *rpoS P*=0.0035) (Fig. 7e)*.* Lower expression of *exoS* is also observed when normalized by *ampC* compared with *rpoS* at 2 h (*ampC* vs. *rpoS P*=0.0037).

## Discussion and conclusions

RT-qPCR is an invaluable technique for assessment of fluctuations in functional gene expression and is commonly used to assess the expression of *P. aeruginosa* infection-associated genes [31,37]. The employment of reference genes is an important normalization step within the RT-qPCR procedure, and the reference genes assessed within this study (*rpoD*, *proC*, *rpoS*, *16S*, *algD*, *gyrA* and *ampC*) are commonly used in the normalization of *P. aeruginosa* during gene expression assays [14,16, 38].

Despite the frequent use of these reference genes, our results show a fluctuation in their expression when *P. aeruginosa* was grown in different media types and across different time points, with *16S* being the only reference gene reproducibly and quantifiably expressed by both strains across all the time points and media types (Figs 1 and 2). This fluctuation in expression across strains, time points and media type challenges traditional assumptions of a reference gene and raises questions regarding the credibility of traditional *P. aeruginosa* reference genes. For *rpoS*, which is predominantly expressed during the stationary growth phase but can also be initiated by stress conditions, including low pH, oxidative stress and increased osmolarity, consideration should be given to growth and environmental conditions when assessing its suitability as a reference gene for gene expression studies [1,39]. For example, a study by Pérez-Osorio *et al.* [40], analysing gene expression per cell in PAO1 biofilms, found the abundance of *rpoS* mRNA to be highest at the top of the biofilms, at the air–biofilm interface, with less than 1 *rpoS* mRNA transcript per cell observed in the middle and base of the biofilm [40]. The same study also found that the levels of *16S* RNA were relatively uniform throughout the biofilm [40]. Similarly to *rpoS*, the expression of *algD* can also be influenced by the external environment, particularly in response to nutrient depletion, oxygen availability and the presence of co-colonizing micro-organisms [41].

Several other studies have observed fluctuations in reference gene expression due to changes in the external environment [1,11]. Meng *et al.* [11] assessed the expression of ten commonly used *P. aeruginosa* reference genes, including *algD*, *gyrA*, *rpoS*, *proC* and *ampC*, upon exposure of *P. aeruginosa* PAO1 to different antibiotic treatments (kanamycin, gentamycin, tetracycline, chloramphenicol, hygromycin, apramycin, tellurite and zeocin). The study, also using the RefFinder algorithms, reported a difference in the reference gene stability profile when *P. aeruginosa* was exposed to antibiotics in comparison to the control group (no antibiotic exposure) [11]. The stability profile also differed between the different antibiotics used, with *algD*, for example, being ranked seventh upon no antibiotic exposure, second when exposed to tetracycline and fourth when exposed to apramycin, tellurite and zeocin [11]. As seen in our results, many of the reference genes in this study exceeded the stability thresholds as depicted by the RefFinder algorithms. As these stability parameters were designed during eukaryotic studies, such threshold cut-offs may be too stringent for bacterial studies, due to the fact that single-cell prokaryotes are much more responsive to a changing environment and generally proliferate much more rapidly than eukaryotes [42]. Thus, more attention should be paid to the reference genes ranking order as opposed to the individual stability values denoted by the programme. In another study by Alqarni *et al.* [1], the stability of 13 *P. aeruginosa* reference genes under carbon starvation (M9 salts without glucose) found that only *rpoS* displayed stable expression at the start of carbon starvation and following 30 min carbon starvation or growth with carbon control. In contrast, *ampC* demonstrated increased levels of mRNA abundance (1.9-fold) following 30 min carbon starvation compared with exposure to carbon during growth [1]. Savli *et al.* [14] also demonstrated lower stability for *ampC* across *P. aeruginosa* strains in comparison to other reference genes, such as *rpoD* and *proC*; however, this study did not assess expression across media types [14,43].

As reference genes are typically used in the normalization of gene expression assays, it seemed appropriate to assess how the employment of differentially ranked reference genes could influence the analysis of a chosen target gene. In this case, *exoS*, a gene responsible for the production of ExoS, a bifunctional type III secretion cytotoxin, was assessed in PAO1 and LESB65 at 2 and 24 h growth time [44] (Figs 6 and 7). In LB, there were no significant differences in *exoS* expression when analysed by *rpoS*, *16S* or *ampC* for both PAO1 and LESB65 at either time point. However, when either PAO1 or LESB65 was grown in SCFM2 and CFLM, there were significant differences identified in *exoS* expression when analysed using the three reference genes. This highlights that, when *P. aeruginosa* is grown in complex media types, the choice of reference gene influences the interpretation of *exoS* expression. For example, when *exoS* expression was normalized with *rpoS* in LESB65 grown for 2 h in CFLM (Fig. 7e), the relative expression of the exotoxin gene was at ~141.0-fold change, compared with 1.38-fold change when analysed by *16S*.

The results of our study, and results reported by other studies [1,11], illustrate that external factors do influence the stability of reference genes, which challenges the traditional notion of a reference gene being constitutively expressed across environments, independent of strain background. This study also highlights that, although the parameters are not appropriate for prokaryotic studies, the RefFinder programme is a useful tool for the ranking of reference genes, and ranking position does influence the interpretation of the target gene when *P. aeruginosa* is grown in complex media.

Although this study identifies *16S* as a reference gene that is expressed in SCFM2 and CFLM across the *P. aeruginosa* PAO1 and LESB65 growth cycle, the limitations of this study must be acknowledged. One such limitation is the focus on only two strains of *P. aeruginosa*. As seen in Figs1  2, the longitudinal expression of the reference genes can fluctuate between strains. To understand these fluctuations further, a larger panel of genetically diverse *P. aeruginosa* strains could be used, with the incorporation of further clinical strains being prioritized. Despite being a dominant micro-organism in several conditions, including CF, *P. aeruginosa* often exists within polymicrobial communities [45]. The presence of co-colonizing micro-organisms can influence *P. aeruginosa* gene expression, and this has been predominantly documented with the presence of Gram-positive bacteria influencing *P. aeruginosa* quorum-sensing gene expression [46,47]. The expression of certain *P. aeruginosa* virulence genes has also been shown to be influenced by *Staphylococcus aureus* in a study by Tognon *et al.* [48], which reports the downregulation of *P. aeruginosa* genes involved in pyochelin synthesis (*pchC*, *pchG*, *pchA*, *pchF*) when grown with *S. aureus* [48]*.* The effects of the polymicrobial community on *P. aeruginosa* reference gene expression specifically have not been studied. This investigation is warranted and will increase understanding of how the expression of *P. aeruginosa* reference genes fluctuates within polymicrobial environments or mixed species infection samples. There is also a risk of non-specific primer amplification when quantifying the expression of *P. aeruginosa* genes within mixed species samples, particularly with *16S*, and therefore primer specificity would need to be checked using both an online tool and against the members of the polymicrobial community being investigated. However, primer specificity within this study has been checked using the NCBI-primer-blast online tool. It must also be noted that other studies have highlighted *16S* as an unreliable RT-qPCR gene in other bacterial species, as demonstrated by Li *et al.* [49] and Tasara and Stephan [50] using *Yersinia enterocolitica* and *Listeria monocytogenes*, respectively*.* However, there are no studies that have reported *16S* as an unreliable reference gene when studying *P. aeruginosa*, and *16S* has recently been employed as a reference gene to study virulence gene expression of *P. aeruginosa* PA14 in host-mimicking conditions [51]. It is also useful to explore whether the *in vitro* expression of a bacterial reference gene accurately reflects its expression within a given infection niche, through comparison with publicly available transcriptomics data. Unfortunately, there are no open-source transcriptomics data available which assess the expression of the *P. aeruginosa 16S* rRNA gene, of which PAO1 has four copies (*PA0668.1*, *PA4280.5*, *PA4690.5* and *PA5369.5*), from CF lung infections. This is likely due to common pre-sequencing steps which intend to remove rRNAs, due to them being the most abundant RNA type in cells [52,53]. This could hinder direct comparative analysis of the expression of *16S* in the CF lung. However, comparisons could be drawn between the expression of *rpoS* and *ampC* in this study and in a study by Lewin *et al.* [52], which uses *Z* scores to assess the effectiveness of several laboratory models to recapitulate the PAO1 transcriptome as it is seen in CF sputum. Lewin *et al.* [52] deem *exoS* expression as ‘accurate’ in PAO1 grown overnight in LB (*Z* score: −0.32364) and SCFM2 (*Z* score: −0.222). *rpoS* expression was also reported to be accurate in LB and SCFM2 (*Z* score: 0.679103 and *Z* score: 0.222, respectively); however, *ampC* expression was ‘accurate’ in LB (*Z* score: −1.60334) and ‘not accurate’ in SCFM2 (*Z* score: −2.3204). The results of this study (Fig. 6b, d) suggest aberrant *ampC* expression in SCFM2, as when *exoS* is normalized using *rpoS* and *16S* in PAO1 at LB 24 h, there are no significant differences in *exoS* expression. However, in SCFM2 at 24 h, there are significant differences in *exoS* expression when analysed with *ampC* in comparison to *16S* and *rpoS*. Here, the relative expression of *exoS* was ~10-fold higher than the control condition when normalized with *16S* and *rpoS*, and 4,000 when normalized using *ampC*. These considerations highlight the importance of assessing reference gene stability prior to initiation of experimental work and tailoring the internal control gene choice to experimental considerations.

This study identifies *16S* as a reference gene that is expressed by *P. aeruginosa* when grown in complex respiratory media across the bacterial growth cycle and highlights its use as an appropriate reference gene for longitudinal *P. aeruginosa* gene expression studies. Although 16S rRNA was the only reference gene consistently expressed by both PAO1 and LESB65 strains across all conditions, the ranking system used in this study has offered valuable insights into the expression profiles of several commonly used * P. aeruginosa* reference genes in complex media. Furthermore, this study highlights the stability of several reference genes, including *rpoS* and *algD*, for studies focusing on later time points (24 and 72 h). This information will be beneficial for future studies that require the use of multiple reference genes for accurate normalization. Furthermore, this work highlights how specific stimuli can influence *P. aeruginosa* gene expression and allows us to assess the impact of environmental interventions on bacterial gene expression, an important consideration for the development of novel antimicrobial therapeutics and antibiotic alternative agents.

## Supplementary material

10.1099/mic.0.001627 Uncited Supplementary Material 1.

## Abbreviations

CF: cystic fibrosis
CFLM: CF lung media
LB: Lysogeny broth
RT-qPCR: quantitative reverse transcription PCR
SCFM2: synthetic CF media 2
